# Efficacy and safety of intrathyroidal dexamethasone therapy in severe subacute thyroiditis – a real-world prospective single center study

**DOI:** 10.3389/fendo.2026.1820684

**Published:** 2026-07-22

**Authors:** Magdalena Stasiak, Magdalena Rosińska, Bartłomiej Stasiak, Renata Michalak, Andrzej Lewiński, Arkadiusz Zygmunt

**Affiliations:** 1 Department of Endocrinology and Metabolic Diseases, Polish Mother’s Memorial Hospital - Research Institute, Lodz, Poland; 2 Institute of Information Technology, Lodz University of Technology, Lodz, Poland; 3 Department of Endocrinology and Metabolic Diseases, Medical University of Lodz, Lodz, Poland

**Keywords:** dexamethasone, intrathyroidal injection, oral prednisone, recurrence, steroid, subacute thyroiditis

## Abstract

**Introduction:**

Clinically severe subacute thyroiditis (SAT) requires high cumulative doses of systemic steroids. The aim of the DLISAT study was to assess efficacy and safety of intrathyroidal dexamethasone and lidocaine injections (DLI) in comparison to standard oral steroid therapy.

**Methods:**

A total number of 60 patients were included in the study, among whom 30 were treated with DLI (study group, SG) and 30 were treated with oral prednisone (control group, CG). In all SG patients, a dose of 4mg of dexamethasone with 1 ml of 2% lidocaine, was injected into each affected thyroid lobe. On the basis of clinical and biochemical results, the doses were repeated every 2–5 days.

**Results:**

Excellent response was observed in all SG patients, with no side effects except for injection-related discomfort. After the first injection, pain decrease and improvement in ESR, CRP, and thyroid parameters were observed. The mean cumulative dose of dexamethasone was 49.7 mg. The mean therapy period was 26.4 days. In CG, the mean therapy period was 118.9 days and the cumulative dose was 255.2 mg (1702.17 mg of prednisone).

**Conclusion:**

DLI was demonstrated to be significantly more beneficial than standard oral steroid therapy, which is especially important for patients with high risk of steroid-related complications.

## 1 Introduction

Subacute thyroiditis (SAT) (also called de Quervain’s thyroiditis, granulomatous thyroiditis or giant cell thyroiditis is a thyroid inflammatory disease of complex pathogenesis. Previous viral infection was demonstrated as a main factor which triggers SAT in genetically predisposed individuals. In Caucasians, genetic susceptibility to SAT was demonstrated to be associated with the presence of HLA-B*35 and C*04:01, as well as of B*18:01, DRB1*01 (1). The alleles HLA-B*18:01 and DRB1*01 were independent SAT risk factors. The highest prevalence of SAT regards middle aged women, and female predominance was widely observed as women constitutes approximately 75%–80% of individuals with the disease (2, 3).

The most common symptom of SAT is anterior neck pain radiating up to the jaw and ear, and down to the upper part of the chest (3–5). However, patients with painless SAT were also described, especially after COVID-19 pandemic (3, 6–8). Interestingly, patients with atypical pain localization, including severe headache, are more and more often reported (9). Fever is often present, and temperatures usually reach over 39°C, rising especially at night. Patients usually present with fatigue and malaise. Symptoms of thyrotoxicosis, include tachyarrhythmias, sweating, tremor, anxiety, insomnia, emotional instability and restlessness. Very high erythrocyte sedimentation rate (ESR) is the most characteristic laboratory finding and C reactive protein (CRP) concentration is also elevated. Biochemical markers of thyrotoxicosis are usually present, but the level of thyroid antibodies is normal in most patients (3–5, 10). The ultrasound (US) pattern of SAT includes hypoechoic and heterogeneous areas with blurred margins, and decreased vascularization (11).

Although the natural course of SAT is self-limiting, most patients complain of severe symptoms, including mainly intense pain, fever and fatigue that deeply worsen quality of life and preclude normal life activities, even for many months.

Mild cases of SAT should be treated with non-steroidal anti-inflammatory drugs (NSAIDs) which are usually efficient in pain and fever control. In patients with more severe SAT, who do not respond to NSAIDs, oral steroids are usually applied. Typical treatment protocol include high initial doses which are gradually decreased throughout several weeks. The main concern of steroid therapy is related to their systemic side effects, with hypertension, edemas, obesity, gastric complications, osteoporosis, hyperglycemia or exacerbation of diabetes, increased susceptibility to infections, muscle weakness, acne, impaired wound healing and psychological instability, including depression (12). It was demonstrated that too early cessation of steroid treatment leads to SAT relapse, and therefore it is difficult to find balance between the risk of recurrence during steroid dose tapering and the risk of complications of long-term steroid therapy. Recurrent course of SAT and steroid dependence lead to high incidence of steroid-related adverse events due to long-term repeated steroid therapy. Other therapies, including even colchicine application were proposed in order to avoid steroid-related complications (13) but their efficacy and safety have not been confirmed.

Recently, subcapsular or supracapsular steroid injection therapy were proposed (14–17). However, due to only a few available publications as well as different steroids and different regimens used by the authors, the actual efficacy and safety of the treatment requires further analysis. Therefore, the aim of the present DLISAT study was to assess efficacy and safety od intrathyroidal dexamethasone injections (DLI) in comparison to standard oral steroid therapy, as well as to provide a useful protocol of such therapy.

## 2 Methods

### 2.1 Patients

This prospective study involved 60 patients treated between November 2023 and August 2025 due to severe SAT, resistant to NSAIDs.

### 2.2 Inclusion criteria

In all patients, the diagnosis of SAT was based on the recently updated criteria (10). The main criteria, both of which should be met, included: elevation of ESR or at least CRP and a presence of hypoechoic area/areas with blurred margin and decreased vascularization in US. The additional criteria, among whom at least one should be met, included hard thyroid swelling and/or pain and tenderness of the thyroid gland/lobe and/or elevation of serum FT4 and suppression of TSH and/or fine needle aspiration biopsy (FNAB) result typical for SAT. The additional criteria include also decreased radioiodine uptake (10), however in our study radioiodine uptake procedure was not performed. FNAB was performed in all doubtful cases in order to exclude malignancy (10, 18).

Severe SAT was defined as a presence of at least one of the following: 1. pain of at least 5 points in NRS scale lasting for at least 5 days; 2. Fever > 38 °C lasting for at least 5 days; 3. CRP ≥ 5 x upper normal limit; 4. ESR ≥ 60 mm/h, with no response to NSAIDs therapy. As the study was performed in a tertiary center, most of patients referred to our center was diagnosed with severe SAT. The total number of patients hospitalized due to SAT in the analyzed period was 84, among whom 21 did not fulfil the criteria of severe SAT and 3 did not agree for any of the offered steroid therapies.

A total number of 60 patients were included in the study, among whom 30 were treated with DLI (study group, SG) and 30 were treated with standard protocol of oral prednisone therapy (control group, CG). Patients in SG were informed that in they could withdraw the consent and change the group any time during the study. None of the patients decided to discontinue DLI.

#### 2.2.1 The inclusion criteria to the SG were as follows

- recent diagnosis of SAT with severe SAT symptoms
- no previous steroid therapy and failure of NSAIDs treatment or recurrence of SAT after oral steroid therapy
- patient’s preference and consent for DLI

#### 2.2.2 The inclusion criteria to the CG were as follows

- recent diagnosis of SAT with severe SAT symptoms
- no previous steroid therapy and failure of NSAIDs treatment or recurrence of SAT after oral steroid therapy
- patient’s preference and consent for oral prednisone therapy

### 2.3 Exclusion criteria

- Lack of patient’s consent for participation in the study
- Current administration of any other medication which may influence treatment outcomes (e.g. substitution therapy with steroids, other anti-inflammatory drugs, biological therapies, anti-viral therapies)
- Previous diagnosis of other diseases which may influence the outcome (e.g. immune deficiencies, acute or chronic inflammatory diseases)
- Hypersensitivity to medications used in the study (dexamethasone or lidocaine)

### 2.4 Study procedures

#### 2.4.1 Study group

**Figure 1:**
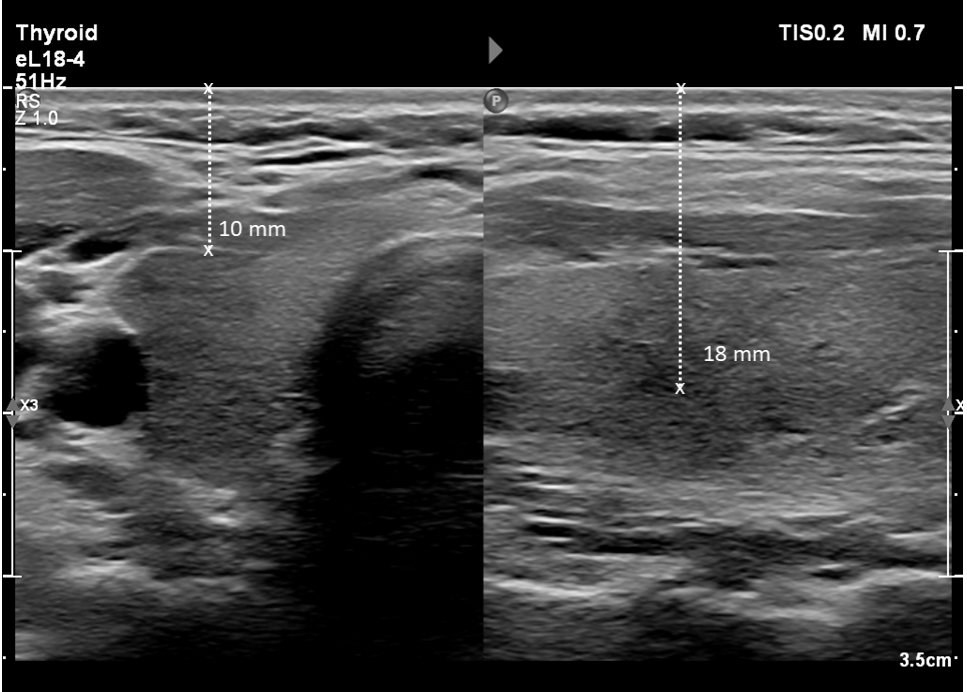
An example of a measurement of distance between the skin and thyroid capsule as well as the skin and the middle of the most affected area of the lobe in a patient of SG.

In all SG patients, a dose of 4 mg of dexamethasone (1 ml of solution), with 1 ml of 2% lidocaine mixed in the same syringe, was injected into each affected thyroid lobe using 22-gauge needle (single injection per lobe). The dose was empirically chosen on the basis of a preliminary tests performed in our center. The choice of needle length was based on the measurement of the mean distance between the skin surface and the anterior thyroid capsule as well as the distance between skin surface and the most affected areas (**Figure 1**). The choice of needle thickness was based on the results of initial trial of different needle size, which revealed that 22-gauge needle is optimal for the procedure due to the fact that the density and stiffness of SAT-affected thyroid areas are high and injections with thinner needles were difficult to perform. The injections were US-guided, and the solution was injected all over the affected lobe, starting from the most painful area and/or area with the highest stiffness in sonoelastography. On the basis of the obtained clinical and biochemical results, doses were repeated every 2–7 days, as required. Before every injection, the following parameters were measured: pain severity, erythrocyte sedimentation rate (ESR), C-reactive protein (CRP), thyroid stimulating hormone (TSH), free thyroxine (FT4), free triiodothyronine (FT3). US pattern and shear wave elastography (SWE) results were applied for diagnosis and for the optimal choice of the main injected area. After every injection, monitoring for complications such as bleeding, hematoma, or recurrent nerve palsy was carried out.

#### 2.4.2 Control group

In all CG patients, prednisone therapy was introduced starting from 40 mg daily. The dose was gradually tapered by 5 mg every week. In patients with symptom recurrence during dose tapering, the dose was increased by 10 mg with further gradual tapering by 5 mg every 2 weeks. In patients with great response to therapy, with complete resolution of symptoms during the first week of therapy, the doses were tapered by 5 mg every 5 days.

### 2.5 Primary and secondary endpoints

The total steroid dose required for complete remission and the therapy duration was considered primary endpoint of the study. Moreover, the treatment time required for significant improvement as well as for normalization of the analyzed parameters in SG group were evaluated. Additionally, frequency of recurrence and of persistent hypothyroidism (i.e. hypothyroidism which lasts 6 months after treatment completion) in SG and CG were compared. Recurrence was defined as re-occurrence of SAT at least 1 month after a complete resolution of the disease and the treatment withdrawal. Persistent hypothyroidism was defined as hypothyroidism which required L-thyroxine therapy at least six months after SAT resolution. The follow-up of the patients was 6–30 months.

### 2.6 Pain severity assessment

Pain severity was assessed with application of NRS, in which a patient rated their pain intensity on a scale of 0 to 10, with 0 meaning “no pain” and 10 meaning the “worst imaginable pain”.

### 2.7 Biochemical procedures

Serum levels of TSH, FT3 and FT4 were measured by an electrochemiluminescence immunoassay (ECLIA) using Cobas e601 analyzer (Roche Diagnostics, USA), ESR was determined with application of Ves-Matic Cube 30 (Diesse, Italy) and CRP was measured using VITROS^®^ 4600 Chemistry System (Ortho Clinical Diagnostics, USA).

### 2.8 Imaging procedures

Ultrasound examinations (US) were performed in every patient, using a 7–14 MHz linear transducer (Philips EPIQ Elite, Philips Ultrasound LLC, Bothell, WA 98021-8431, USA). In order to find the most appropriate area for DLI, the extent, location and US pattern and shear wave stiffness (SWS) of SAT-related lesions were examined.

### 2.9 Cytological procedures

In all doubtful cases, fine needle aspiration biopsies (FNAB) were performed using a 23-gauge needle. Smears were cytologically evaluated, and the presence of multinucleated giant cells together with mononucleated macrophages and of follicular epithelial cells present on an acute and chronic inflammatory dirty background (comprising of cellular debris and mixed inflammatory cells) constituted cytological confirmation of SAT.

### 2.10 Statistical analysis

Descriptive statistics of the collected material comprised mean, standard deviation and order statistics: min, median, max and interquartile range (IQR). For comparisons between the study group and the control group (**Table 1**) Mann-Whitney U test was used as all the parameters were non-normally distributed (which was confirmed by Shapiro-Wilk normality test). In order to evaluate the patients’ outcomes after individual doses (**Table 2**) paired tests (for dependent samples) were used. t-Student paired test was used for normally distributed differences (mostly typical for the ESR parameter) and Wilcoxon signed rank (paired difference) test - in the case of non-normal distribution (as verified by preliminary Shapiro-Wilk test). For binary outcomes (SAT recurrence and persistent hypothyroidism) presented in **Table 3** we used chi2 test and the treatment time and dexamethasone dose were compared between SG and CG with Mann-Whitney U test. All numerical processing was performed with the aid of scipy library (version 1.10.1) for Python programming language (version 3.8.10).

**Table 1:** Comparison of laboratory test results obtained before treatment between SG and CG.

Parameter (unit) (reference range)	Group	Mean	SD	Median	Minimum	Maximum	IQR	*p*-value
ESR (mm/h) (<12)	SG	78.10	35.361	83	22	140	63.750	0.596
	CG	71.000	20.315	72	22	117	22.750	
CRP (mg/dl) (< 1.0)	SG	6.799	7.960	4.355	0.500	33.800	6.715	0.487
	CG	6.740	6.925	4.870	1.080	32.980	3.987	
TSH (mIU/l) (0.27-4.2)	SG	0.571	0.844	0.030	0.005	2.600	1.323	0.116
	CG	0.185	0.478	0.010	0.005	2.350	0.030	
FT4 (ng/dl) (0.93-1.7)	SG	2.806	1.639	2.595	0.980	7.030	2.327	0.877
	CG	2.633	1.229	2.240	1.170	6.740	1.385	
FT3 (pg/ml) (2.0-4.4)	SG	5.808	3.720	4.445	1.480	16.390	3.958	0.171
	CG	6.471	3.328	5.445	3.160	17.250	4.552	
Anti-TPO (IU/ml) (< 34)	SG	38.376	70.515	12.300	4.800	269.500	10.700	0.850
	CG	24.188	33.431	12.245	5.450	157.500	10.442	
Anti-Tg (IU/ml) (<115)	SG	205.055	338.61	13.390	43.700	1552.000	259.00	0.175
	CG	110.778	176.67	37.845	3.230	765.100	96.195	

**Table 2:** Changes in biochemical parameters and pain before DLI and after subsequent doses of DLI therapy up to 7^th^ dose and at the end of treatment (EoT) visit.

Parameter (unit) (reference range)	Treatment point	Mean	SD	Median	Minimum	Maximum	IQR	p-value
Pain	Before	6.000	1.145	6.000	4.000	8.000	2.000	–
	Dose #1	3.310	0.891	3.000	2.000	5.000	1.000	0.000
	Dose #2	1.690	1.004	2.000	0.000	4.000	1.000	0.000
	Dose #3	0.893	0.685	1.000	0.000	3.000	0.250	0.000
	Dose #4	0.769	0.863	0.500	0.000	2.000	1.750	0.000
	Dose #5	0.375	0.647	0.000	0.000	2.000	1.000	0.000
	Dose #6	0.400	0.598	0.000	0.000	2.000	1.000	0.000
	Dose #7	0.067	0.258	0.000	0.000	1.000	0.000	0.000
	EoT	0.000	0.000	0.000	0.000	0.000	0.000	0.000
ESR	Before	78.889	36.506	90.000	22.000	140.000	66.000	–
	Dose #1	71.207	40.550	74.000	12.000	140.000	73.000	0.040
	Dose #2	45.214	31.335	32.500	2.000	106.000	46.000	0.000
	Dose #3	33.536	27.573	26.500	2.000	123.000	25.750	0.000
	Dose #4	33.000	20.471	31.000	5.000	78.000	29.250	0.000
	Dose #5	22.826	14.850	15.000	2.000	53.000	22.000	0.000
	Dose #6	23.300	16.416	21.500	1.000	61.000	22.000	0.000
	Dose #7	16.200	10.359	13.000	2.000	36.000	14.000	0.000
	EoT	9.667	7.401	8.000	1.000	30.000	10.500	0.000
CRP	Before	7.120	8.137	4.625	0.500	33.800	6.942	–
	Dose #1	3.415	3.320	1.650	0.500	12.280	3.903	0.000
	Dose #2	1.973	2.722	0.975	0.500	11.350	1.307	0.000
	Dose #3	2.460	3.190	0.920	0.500	13.030	1.820	0.001
	Dose #4	2.451	3.002	1.165	0.500	12.770	2.165	0.002
	Dose #5	1.440	1.717	0.850	0.500	8.000	0.825	0.000
	Dose #6	1.465	1.138	1.075	0.500	4.710	1.712	0.000
	Dose #7	0.842	0.537	0.500	0.500	2.060	0.500	0.000
	EoT	0.846	1.093	0.500	0.050	5.940	0.002	0.000
TSH	Before	0.570	0.850	0.030	0.005	2.600	1.305	–
	Dose #1	0.619	1.122	0.030	0.005	3.700	0.520	0.494
	Dose #2	1.028	1.753	0.060	0.005	7.240	1.505	0.019
	Dose #3	1.286	1.823	0.175	0.005	6.180	1.867	0.001
	Dose #4	1.338	2.231	0.285	0.005	8.650	1.557	0.001
	Dose #5	2.114	3.880	1.380	0.005	18.940	1.955	0.001
	Dose #6	4.137	9.736	1.385	0.005	44.680	3.670	0.001
	Dose #7	9.227	25.241	2.930	0.005	100.000	3.620	0.001
	EoT	2.606	1.994	2.000	0.010	7.370	2.765	0.000
FT4	Before	2.742	1.618	2.570	0.980	7.030	2.285	–
	Dose #1	2.573	1.398	2.420	0.890	5.260	2.130	0.005
	Dose #2	2.254	1.198	2.015	0.960	5.730	1.427	0.023
	Dose #3	1.860	1.084	1.625	0.610	5.650	0.708	0.000
	Dose #4	1.590	0.786	1.355	0.810	4.100	0.585	0.000
	Dose #5	1.394	0.537	1.315	0.660	2.540	0.662	0.001
	Dose #6	1.241	0.469	1.140	0.410	2.350	0.367	0.000
	Dose #7	1.254	0.550	1.120	0.690	2.810	0.420	0.000
	EoT	1.073	0.231	1.060	0.610	1.430	0.380	0.000
FT3	Before	5.467	3.278	4.340	1.480	15.990	3.895	–
	Dose #1	4.554	2.446	3.650	1.160	10.290	3.810	0.000
	Dose #2	4.396	1.932	4.135	1.570	9.670	2.658	0.001
	Dose #3	3.781	2.011	3.190	1.840	12.000	1.147	0.001
	Dose #4	3.492	1.385	3.255	1.830	8.490	0.950	0.000
	Dose #5	3.112	1.288	3.120	0.720	5.950	0.880	0.001
	Dose #6	2.936	0.969	2.975	0.620	5.000	0.547	0.000
	Dose #7	2.831	0.998	2.650	0.750	4.580	0.965	0.003
	EoT	3.002	0.476	3.070	1.830	3.940	0.480	0.001

Abbreviations: EoT, end of treatment; IQR, interquartile range; SD, standard deviation.

**Table 3:** Comparison of treatment duration, drug dose, no. of cases with recurrence and no. of cases with permanent hypothyroidism between SG and CG.

Parameter (unit)	Group	Mean	SD	Median	Minimum	Maximum	IQR	p-value
Treatment time (days)	SG	26.4	11.056	27	4	43	15.000	<0.0001
	CG	118.9	60.546	90	60	335	33.500	
Dexamethasone dose (mg)	SG	49.7	20.789	52	8	96	28.000	<0.0001
	CG	255.2	146.44	190	143	864	74.750	
Parameter	Group	Result			p-value			
Recurrence (No. of cases)	SG	3			0.923			
	CG	3						
Persistent hypothyroidism (No of cases)	SG	3			0.552			
	CG	5		

### 2.11 Ethics procedures

The study was conducted in accordance with the Declaration of Helsinki. Informed consent for all performed procedures was obtained from all of the patients after a full explanation of the purpose and nature of all procedures used. The study was approved by the Ethics Committee of the Polish Mother’s Memorial Hospital—Research Institute, Lodz, Poland (Project identification code – 73/2022).

## 3 Results

The mean age of patients in the analyzed groups was 45.83 ± 10.69 and 42.23 ± 8.75 years in SG and CG, respectively (p=0.159). The male/female ratio was 3/27 in both groups. No difference between SG and CG with regard to pre-treatment laboratory results, including inflammatory parameters, was found (**Table 1**).

In SG patients, the mean distance between the skin surface and the anterior thyroid capsule was 7.5 ± 2,02 mm, median 7 mm, while the distance between skin surface and the most affected areas was 16.43 ± 4.84 mm, median 16 mm (**Figure 1**).

**Figure 2:**
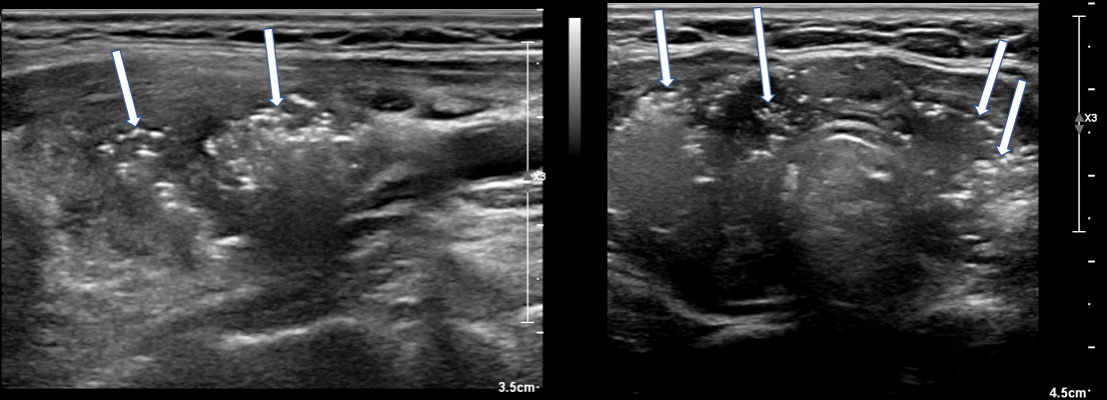
Ultrasound pattern of the thyroid lobes directly after intrathyroidal DLI. Arrows indicate characteristic snow-like effects demonstrating the spread of DLI in the affected areas.

The injections were US-guided, and the solution was injected all over the affected lobe, starting from the most painful area and/or area with the highest stiffness in sonoelastography. The control of the proper distribution of the drug in the thyroid is demonstrated in **Figure 2**.

**Figure 3:**
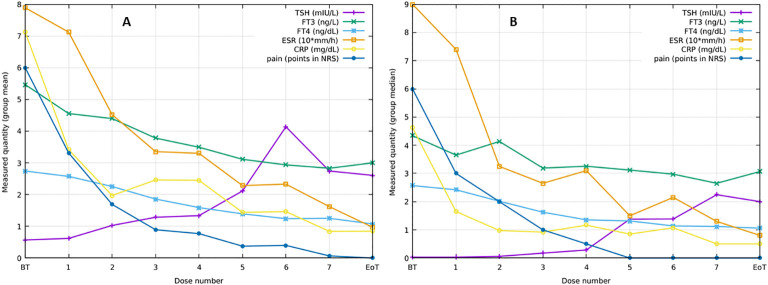
Changes in the mean **(A)** and median **(B)** values of the measured parameters after each of the consecutive doses in the study group (SG). BT - before treatment, EoT - end of treatment. TSH mean value and median value was presented for 29 patients, after exclusion of a patient with severe persistent hypothyroidism. An increase in TSH level illustrates transient hypothyroidism, most frequently visible after 6th dose of DLI.

Excellent rapid and long-term response was observed in all SG patients, with no side effects except for injection-related discomfort. After the first injection, all SG patients reported immediate pain decrease from 4–8 to 2–5 points in the numeric rating scale (NRS) (**Table 2**). Similarly, an improvement in ESR, CRP, and thyroid parameters was observed. **Table 2** presents gradual normalization of biochemical parameters after subsequent doses. In the case of all parameters except for TSH, statistically significant difference was achieved already after the first dose (**Table 2**). Rapid and long term improvement of all the analyzed parameters with clear presence of typical transient hypothyroid state are visible in **Figure 3**.

Due to the impact of one case of severe hypothyroidism, which heavily influenced the mean value of TSH after dose 6th and 7th, this single patient was excluded from the data used to generate the mean TSH plot in **Figure 3**. The related outlier values are, however, well visible in **Table 2**: the maximum and mean TSH after the 7^th^ dose are 100 and 9.227, respectively, while excluding this patient reduces the mean TSH after the 7^th^ dose down to 2.9 mIU/l, as evidenced in **Figure 3** (naturally, the median value stays practically unchanged).

The mean number of doses required for total recovery was 6,83 ± 2,88, median 7. The number of doses given to patients were as follows: 1 (1 patient), 2 (1 patient), 3 (2 patients), 4 (2 patients), 5 (2 patients), 6 (5 patients), 7 (8 patients), 8 (2 patients), 9 (2 patients), 10 (2 patients), 12 (1 patient), 13 (2 patients). Nine patients required more than 7 injections. Among this group there were 3 patients with SAT recurrence after oral therapy in the course of steroid-dependent disease.

A mean dose of steroid required for a total cure was significantly lower and treatment duration was significantly shorter in SG than in CG. A cumulative mean dose of dexamethasone for the total therapy was 49.7 mg in SG, while in CG a mean dose of oral prednisone was 1702.17 mg, which is equivalent to 255.2 mg of dexamethasone (using a well-known conversion factor of 6.67, i.e. 1 mg of dexamethasone = 6.67 mg of prednisone). The longest therapy period in SG was 43 days. The frequency of recurrence and permanent hypothyroidism was similar in both groups. Three cases (10%) of recurrence were observed in both groups. Persistent hypothyroidism occurred in three patients (10%) of SG and in 5 cases (16.67%) of CG. Comparison of efficacy of DLI and oral prednisone therapy with regard to treatment duration, steroid dose, risk of recurrence and risk of persistent hypothyroidism is presented in **Table 3**.

## 4 Discussion

Although SAT is often a self-limiting disease, many patients suffer from severe symptoms which may impede normal life activities even for many weeks. Therefore, anti-inflammatory therapy constitutes a standard management of SAT. Severe cases of SAT, not responding to NSAIDs therapy, require steroid-based treatment. The most frequently applied oral therapy regimen includes prednisolone (PSL) at the dose of 40 mg daily for 1–2 weeks followed by a gradual dose tapering over 4–12 weeks or longer (3, 18–20). Adverse effects of such long and/or high-dose steroid therapies include weight gain, hypertension, hyperglycemia, edemas, gastritis, osteoporosis, infections, acne, depression and many others. In order to avoid oral steroid-related complications, other therapy modalities for SAT are being evaluated. In the observational study by Kubota et al., PSL at the daily dose of 15 mg initially, with further tapering by 5 mg every 2 weeks was efficient and safe in Japanese cohort (21). However, it was demonstrated that over 20% of patients with SAT require more than 8 weeks of therapy (15). Therefore, it seems crucial to establish the method of therapy which is efficient without exposure of patients to high doses and long-term steroid therapy.

In the present study, we have demonstrated that the efficacy and safety of intrathyroidal injections of dexamethasone and lidocaine are superior to standard oral therapy with regard to the cumulative steroid dose, therapy duration, and the speed of pain relief and normalization of inflammatory and thyroid parameters. The mean therapy duration was significantly shorter and the cumulative steroid dose was significantly lower in SG as compared to CG.

To the best of our knowledge, this is the first study which demonstrated superiority of intrathyroidal DLI in efficacy and safety of treatment as compared to standard oral therapy in patients with severe SAT with no other additional therapy. Previous studies included intrathyroidal injections of triamcinolone and betamethasone or capsular injections of dexamethasone with additional NSAIDs therapy in the cases with persistent symptoms (15–18). The choice of the most optimal method and protocol of the procedure seems to be crucial. Therefore, our protocol and results should be compared to the previous ones. Hu et al. (15) demonstrated significant efficacy of triamcinolone with lidocaine injected with insulin pen. This study included the largest cohort already studied (93 participants). However, significant differences between our study and the study by Hu et al. should be taken into account. Firstly, the authors declared that due to expansion of the thyroid swelling toward the skin, the distance from the skin to the thyroid capsule was diminished to 2–3 mm in the isthmus and to 3–5 mm in the thyroid lobes, therefore the pathological areas (hypoechoic ones) were injected with 4-mm needle and 6–8 mm needle in the case of isthmus and lobes, respectively. In our study, the distance between the skin surface and thyroid capsule was more than twice longer, i.e. 5–12 mm, than the one reported by Heu et al. (15) (**Figure 2**). Moreover, the mean distance between the skin and SAT-related lesions in the thyroid lobe was 16.43 mm. Therefore, a method based on insulin pen was entirely not applicable in our cohort. The discrepancies in the described distance values may be related to the study population and difference in neck morphology between Asian and European cohorts. Secondly, the protocol by Hu et al. included administration of 3 injections daily for the first three days, and then 3 injections daily every other day. If pain was present at the end of the first week of therapy, oral ibuprofen was added to the regimen and if SAT symptoms persisted or recurred after two weeks of therapy, prednisone or ibuprofen was administered orally (15). In our study, DLI was applied as the only therapy in SG, in order to evaluate an actual cumulative dose of dexamethasone and an actual therapy duration from the beginning of the treatment to SAT cessation. Moreover, the consecutive doses were applied only if required, so the patients received different number of doses. Therefore, we cannot directly compare our doses or treatment duration with the results obtained by Hu et al. The final results of the authors’ study (15) are also difficult to compare with ours, as in our study no other anti-inflammatory medication was added throughout the study period. Patients who took NSAIDs or oral steroids during DLI therapy were not included into the present analysis.

Forkert et al. (17) performed a study similar to ours with regard to comparison of a group treated with intrathyroidal injections and with oral prednisone. Control group received a course of prednisone 20 mg per day for 4 weeks. The study group received a preparation containing 6,43 mg of betamethasone dipropionate micronized and 2,63 mg of betamethasone sodium phosphate mixed with 2% 1 ml lidocaine in a syringe with 12 mm needle. The authors gave the injections only twice: at baseline and at two week visit. However, the injections were repeated during one session and the solution was injected evenly into the thyroid (both lobes and the isthmus) with a 10 mm distance between injections. The authors observed definitely faster result of intrathyroidal injections as compared to systemic steroids. After eighth week of the study they observed 3 cases of relapse in oral steroid group and one case of relapse in injection group (17). This is a very interesting study, especially because the authors claimed that only two injection procedures were sufficient, while the median number of injections in our study was 7. The reasons of such differences between studies may be complex because the initial severity of inflammatory parameters (ERS, CRP) was much lower in the quoted study than in ours (17). Small group size (16 patients in each group) may also have an impact on the results. However, an impressive result obtained by the authors may indicate that a preparation containing micronized betamethasone dipropionate and betamethasone sodium phosphate may be more efficient than other steroids in patients with low grade severity of SAT.

The first report on efficacy of dexamethasone injections (14), presented application of the drug together with lidocaine with an insulin pen, using 4 mm needle for an isthmus and 6 mm needle for the lobes. We also believe that the dose administered to the lobe is difficult to compare as 6 mm needle could allow probably only for sub or supracapsular application, while in our study DLI was administered into the most affected thyroid areas. Ma et al. confirmed better efficacy of the therapy as compared to oral steroids. The injections were administered every other day and from the second week ibuprofen was added. Therefore, once again, the comparison of this study with ours is not possible, as in our study NSAIDs application was not allowed.

Similar study was performed by Huo et al. (16) who compared efficacy of dexamethasone injections given into thyroid capsule every other day for one week (3 sessions) and of oral prednisone in an initial dose of 20 mg. A patient of the study group was considered clinically cured if the pain did not recur 72 hours after treatment discontinuation. Patients with persistent pain after the third session injections received aspirin at a dose of 600 mg/day. Unfortunately, the basal ESR is not available, and the first follow up results are after 4 weeks, and therefore, the results are also not comparable to ours.

It should also be noticed that in the cases of studies with capsular or subcapsular steroid injections, the probability of administration of the drug into blood vessels (or fast elimination of the drug by blood vessels) is high, as these are vessels-abundant areas. Moreover, no trace of the drug can be visible in the US examination of the thyroid. In our study, we could see the “snow-like” trace of the drug inside the injected lobes, to confirm the optimal administration of the drug (**Figure 3**). Therefore, intrathyroidal injections seem to provide more stable and more easily controlled application of the drug, which is especially important in patients with severe SAT. Nevertheless, all the previously performed trials with capsular, supracapsular and intrathyroidal injections of different steroids presented superiority of those methods with respect to the classic oral regimen.

In the present study, the application of DLI provided rapid pain relief after the first dose and fast normalization of inflammatory and thyroid parameters. The cumulative dose of steroid was 5 times lower in SG than in CG group and the therapy duration was almost 5 times shorter. The method with 22G needle allows for precise administration of DLI into affected areas and the drug spread is clearly visible as a “snow-like” ultrasound pattern. Our study also confirmed that in severe SAT, the number and frequency of doses should be optimally based on the clinical and biochemical parameters and adjusted individually to each patient as the number of doses required for total recovery are very diverse. In our cohort, four patients with relapse due to steroid-dependent course of the disease were successfully treated, although it should be noted, that they constituted the group who required the largest number of doses and the longest therapy duration.

In conclusion, our study demonstrated the high efficacy and safety of DLI therapy in Caucasian population with severe SAT, with highly elevated inflammatory markers, non-responsive to NSAIDS. Due to the lower steroid dose and short treatment time, DLI was proved to be significantly more beneficial than standard oral steroid therapy and this therapy protocol can be recommended in all patients with severe SAT in order to avoid steroid-related adverse effects. The proposed method can constitute an optimal solution and seems to be crucial for patients with high risk of steroid-related complications as well as with steroid dependent SAT course.

## Data Availability

The original contributions presented in the study are included in the article/supplementary material. Further inquiries can be directed to the corresponding author/s.
